# Bilious Vomiting and Volvulus: The Eyes Cannot See What the Mind Does Not Know

**Published:** 2011-03-10

**Authors:** Abid Qazi

**Affiliations:** Department of Paediatric Surgery, Leeds General Infirmary, United Kingdom LS1 3EX

**Dear Sir**

Neonates referred from various sources with single or more episodes of bilious vomiting undergo radiological investigation and ultrasound examination to exclude malrotation and volvulus. Although many of these will have a normal study and feed well afterwards or may have other diagnoses, nevertheless, approximately 6 to 8% will need corrective surgery for malrotation [1, 2]. Rarely an older child may present with similar symptoms.

A study suggested that even in developed world bilious vomiting might go unrecognized by medical professionals and parents. Green colour vomiting is single most important symptom, which should lead to further investigations [3]. It is expected that in Pakistan where health care facilities are not well organized and awareness about paediatric surgical diseases is lacking, a significant hidden morbidity and mortality may be associated with this condition.

The consequences of missed or delayed diagnosis can be catastrophic, leading to long-term morbidity or mortality. A typical newborn with malrotation and volvulus present with bright green vomiting (Fig. 1). It may be the only symptom in early phase. At this stage bowel may be completely viable. Later symptoms can be abdominal distention, sudden collapse due to haemodynamic instability or bleeding per rectum. A plain x-ray may be suggestive due to unequal distribution of bowel gas. However a normal x-ray does not rule out presence of volvulus. An upper GI contrast with real time imaging by an expert radiologist is quite diagnostic. Elements of radiological diagnosis are position of duodenojejunal junction in relation to spine. An obstructed or corkscrew duodenum is suggestive of the presence of volvulus. A contrast enema has been used in the past to assess caecal position. If caecum is high and fixed towards the midline, an indirect inference can be deducted about the presence of malrotation. However a normal caecal position cannot exclude malrotation. If in doubt ultrasound imaging is usually complemented with contrast study to assess the orientation of mesenteric vessels. Superior mesenteric artery to the right of vein or a whirl pool appearance is suggestive of malrotation and volvulus, respectively.

**Figure:**
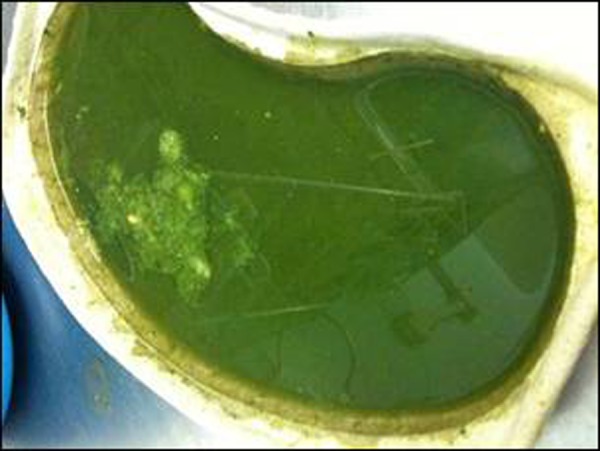
Figure 1: Typical dark green vomit often referred to as bilious vomiting

An unstable child with bilious vomiting and obvious abdominal signs should not undergo any further investigations as prompt surgical intervention is crucial to save ischemic bowel. There is an apparent cost of performing contrast study in majority of normal neonates but comparing it to the cost of catastrophic loss of bowel, long term requirement of parenteral nutrition, ongoing surgical care, prolonged hospital stay and loss of life is much more. Some of these late diagnosed children may even require bowel and liver transplantation.

It is extremely important for all clinicians, nurses and midwives to recognize bilious vomiting and understand that green vomiting means mechanical obstruction unless proved otherwise. It is our responsibility as paediatric surgeons to spread the message to paediatricians, obstetricians, midwives and general practitioners to identify the bilious vomiting and significance of delay in diagnosing volvulus.

## Footnotes

**Source of Support:** Nil

**Conflict of Interest:** None declared
